# A Novel Selective Axl/Mer/CSF1R Kinase Inhibitor as a Cancer Immunotherapeutic Agent Targeting Both Immune and Tumor Cells in the Tumor Microenvironment

**DOI:** 10.3390/cancers14194821

**Published:** 2022-10-02

**Authors:** Yeejin Jeon, Hwankyu Kang, Yeongin Yang, Dongsik Park, Baejung Choi, Jeongjun Kim, Jaeseung Kim, Kiyean Nam

**Affiliations:** Qurient Co., Ltd., Seongnam-si 13487, Korea

**Keywords:** Axl, Mer, CSF1R, immune checkpoint, tumor microenvironment, immune evasion, PD-1, small molecule inhibitors, cancer

## Abstract

**Simple Summary:**

Immune checkpoint blockade has had great success over the past decade, but many patients with cancer do not benefit because most immune checkpoint inhibitors only target T cells. Targeting non-T cell populations in the tumor microenvironment (TME) has been shown to affect responses to them. Simultaneous inhibition of Axl, Mer and CSF1R by a novel receptor tyrosine kinase inhibitor Q702 induces antitumor immunity by reducing the number of M2 macrophages and MDSCs and inducing M1 macrophages and cytotoxic CD8 T cells in the TME, and increasing the expression of MHC-I and E-cadherin in tumor cells. Our data indicate that therapy targeting both immune cells and cancer cells in the TME by Q702 can induce more effective clinical responses in patients with cancer.

**Abstract:**

Although immune checkpoint blockade (ICB) represents a major breakthrough in cancer immunotherapy, only a limited number of patients with cancer benefit from ICB-based immunotherapy because most immune checkpoint inhibitors (ICIs) target only T cell activation. Therefore, targeting non-T cell components in the tumor microenvironment (TME) can help subvert resistance and increase the applications of ICB-based therapy. Axl and Mer are involved in the carcinogenesis of multiple types of cancer by modulating immune and biological behaviors within tumors. Colony stimulating factor 1 receptor (CSF1R) mediates tumorigenesis in the TME by enhancing tumor associated macrophage (TAM) and myeloid-derived suppressor cell (MDSC) infiltration, facilitating immune escape. Therefore, the simultaneous inhibition of Axl, Mer, and CSF1R kinases may improve therapeutic efficacy by targeting non-T cell components in the TME. Here, we present Q702, a selective, potent small molecule inhibitor targeting Axl, Mer, and CSF1R, for oral administration. Q702 induced antitumor activity in syngeneic tumor mouse models by: remodeling the TME toward immune stimulation; expanding M1 macrophage and CD8 T cell populations and decreasing M2 macrophage and MDSC populations in the TME; and increasing MHC class I and E-cadherin expression in tumor cells. Thus, Q702 may have great potential to broaden the coverage of populations benefiting from ICB-based immunotherapy.

## 1. Introduction

Cancer cells often hijack the mechanisms that maintain immune homeostasis to evade the immune system, such as the programmed death protein 1/programmed cell death ligand 1 (PD-1/PD-L1) signaling pathway. Increased PD-L1 expression in tumor cells suppresses T cell activity, thereby downregulating the immune response [1]. In addition, cancer cells often use specific mechanisms that prevent T cells from perceiving imminent threat [2]. Therefore, the restoration of immune homeostasis is considered an important immunotherapeutic approach for cancer, and the development of anti-PD-1 or anti-PD-L1 antibodies has helped to improve the long-term survival of patients with various solid tumors [3]. Nevertheless, most patients with cancer do not benefit from treatment with current T cell checkpoint inhibitors because of the suppressive milieu in the tumor microenvironment (TME), which comprises suppressive innate immune cells, including myeloid-derived suppressor cells (MDSCs), M2-like macrophages, and tumor-associated macrophages (TAMs) [4,5]. The suppression of innate immune cells leads to the failure of T cell recruitment to tumor tissue and/or suppression of T cell activity, which facilitates immune system evasion by cancer cells. Immune evasion by cancer cells is not limited to the suppression of immune functions but also occurs via the downregulation of the antigen presentation capacity of cancer cells [6]. Therefore, the restoration of innate immune homeostasis and tumor antigen presentation capacity can be an effective strategy for improving current cancer immunotherapies involving T cell checkpoint inhibitors.

Axl, Mer, and the colony stimulating factor 1 receptor (CSF1R) are key molecules involved in innate immune homeostasis and antigen presentation. These are transmembrane proteins of receptor tyrosine kinases. Axl is expressed in cells of hematopoietic and non-hematopoietic lineages, whereas Mer and CSF1R are preferentially expressed in cells of hematopoietic lineages, such as monocytes/macrophages. Axl activation is directly associated with tumor cell survival, anti-apoptotic signaling, mitogenesis, migration, invasion, drug resistance, and especially epithelial-mesenchymal transition (EMT) [7]. Whether Axl expression triggers EMT or whether EMT induces Axl expression remains controversial; however, evidence from several mechanistic studies supports the hypothesis that Axl is an EMT inducer. A kinome-wide shRNA screen also identified Axl as a key regulator of the mesenchymal state and stem cell properties in glioblastoma [8]. Moreover, a study using breast cancer models showed that Axl downregulation could reverse the EMT phenotype in cancer stem cell populations [9]. EMT, one of the major molecular mechanisms involved in oncogenesis, promotes cancer progression. In addition, it is a crucial step in which cancer cells acquire the ability to evade the immune system. Low major histocompatibility complex class I (MHC-I) expression has been reported in tumor cells undergoing EMT, which reduces their antigen presentation potential and consequently attenuates T cell-mediated lysis [10]. Therefore, EMT may trigger a cascade of events that ultimately lead to immunosuppression, and Axl may serve as a key regulator of this process.

The receptor tyrosine kinase Mer plays a prominent role in the regulation of innate immunity by a regulatory feedback mechanism that limits the extent of the inflammatory response. Mer is expressed in myeloid cells, especially macrophages, because it is essential for the efferocytosis of apoptotic cells. Mer binds to phosphatidylserine (PS), an “eat-me” signaling molecule, on apoptotic cells via its bridging ligands, growth arrest specific 6 (Gas6) or Protein S (ProS), and induces phagocytosis, known as “efferocytosis” [11]. When macrophages engulf apoptotic cells through Mer, they polarize to the M2 phenotype, alter immune metabolism, and secrete immunosuppressive cytokines, which are important steps for maintaining immune homeostasis, thereby preventing chronic inflammation and autoimmunity. However, in cancer, the environment induced by macrophages through efferocytosis is similar to the immunosuppressive phenotype of the TME. Thus, cancers employ TAMs with the M2 phenotype via Mer to suppress the innate immune sensing of tumors [12,13].

CSF1R, also known as macrophage colony-stimulating factor receptor, is another key regulator of macrophage polarization as CSF1R-mediated signaling is important for the growth, proliferation, survival, differentiation, and function of macrophages and other myeloid cells, including MDSCs [14]. Sustained CSF1R activation by its ligands in the TME results in polarization of the M2 TAM phenotype and promotes tumor progression, inhibiting immune-stimulatory signals; therefore, it has been considered as a promising therapeutic target [15]. However, CSF1R inhibitors have not shown clinical success in monotherapy due to their limited efficacy, which is associated with the accumulation of polymorphonuclear MDSCs (PMN-MDSCs) following CSF1R inhibition. Nevertheless, CSF1R remains a promising target for immunotherapy that can markedly improve the efficacy of T cell checkpoint immunotherapy and lead to tumor regression because CSF1/CSF1R inhibition decreases the number of TAMs and reduces immune suppression by reprogramming the remaining TAMs to support antigen presentation and enhance T cell activation within the TME [16,17,18].

In this study, we document the characterization of the Axl/Mer/CSF1R inhibitor, Q702, in cancer immunotherapy. In addition, we demonstrate that the simultaneous inhibition of Axl, Mer, and CSF1R induces antitumor effects by altering the immune profile and cancer cell phenotype in the TME. The findings of this study suggest that therapy targeting both immune cells and cancer cells in the TME by a novel small molecule inhibitor, Q702, can induce more effective clinical responses in patients. 

## 2. Materials and Methods

### 2.1. Cell Lines and Reagents

Cell lines were purchased as follows: H1299, A549, THP-1, M-NSF-60, EMT6, CT26, and RENCA (ATCC, Manassas, VA, USA), B16F10-OVA (Crownbio, San Diego, CA, USA), MC38 (BioVector NTCC Inc., Beijing, China), and all cells were confirmed to be pathogen-free (including Mycoplasma testing; Lonza; #L108-318). Q702 was synthesized using Qurient Co., Ltd. (Seongnam-si, Korea). Anti-PD-1 antibody was purchased from Bio X Cell (West Lebanon, NH, USA; #BE0146, clone RMP1-14). The following antibodies were used in western blotting: phospho-Axl-(Tyr702) (Cell Signaling Technology, Danvers, MA, USA; #5724, clone D12B2), Axl (Cell Signaling Technology; #8661, clone C89E7), phospho-AKT(Ser473) (Cell Signaling Technology; #4060S, clone D9E), AKT (Cell Signaling Technology; #9272S), phospho-Mer(Tyr749/Tyr753/Tyr754) (Abcam, Cambridge, UK; ab14921), Mer (Cell Signaling Technology; #4319, clone D21F11), phospho-CSF1R(Tyr723) (Cell Signaling Technology; #3155S, clone 49C10), CSF1R (Cell Signaling Technology; #3152S), phospho-ERK1/2(Thr202/Tyr204) (Cell Signaling Technology; #4370S, clone D13.14.4E), ERK1/2 (Cell Signaling Technology; #4695S, clone 137F5), and β-actin (Sigma-Aldrich, St. Louis, MO, USA; #A5441). PMA was purchased from Sigma-Aldrich (#P1585), and ionomycin calcium salt was purchased from Tocris Bioscience (Bristol, UK; #1704).

### 2.2. Enzyme Binding Assay for Axl, Mer, and CSF1R

The assay was performed using DiscoverX (KINOMEscan^TM^ Profiling; Luxembourg). Kinase-tagged T7 phage strains were prepared from *E. coli* derived from the BL21 strain. *E. coli* cells were grown to the log phase, infected with T7 phage, and incubated under shaking conditions at 32 °C until lysis. Lysates were centrifuged and filtered to remove cell debris. The remaining kinases were produced in HEK293 cells and were subsequently tagged with DNA for qPCR. Streptavidin-coated magnetic beads were treated with biotinylated small-molecule ligands for 30 min at room temperature to generate affinity resins for the kinase assays. The ligand-attached beads were blocked with excess biotin and washed with a blocking buffer (SeaBlock, 1% BSA, 0.05% Tween 20, and 1 mM DTT) to remove unbound ligands and reduce non-specific binding. Binding reactions were conducted by combining kinases, ligand-attached affinity beads, and test compounds in a binding buffer (20% SeaBlock, 0.17 × PBS, 0.05% Tween-20, and 6 mM DTT). Q702 was serially diluted and transferred to a polypropylene 384-well plate for 1 h at room temperature with shaking, and the affinity beads were washed with a wash buffer (1× PBS and 0.05% Tween-20). The beads were then resuspended in elution buffer (1× PBS, 0.05% Tween-20, and 0.5 µM non-biotinylated affinity ligand) and incubated at room temperature for 30 min with shaking. The kinase concentrations in the eluates were measured by qPCR. The binding constant (Kd) was calculated from a standard dose-response curve using the Hill equation:

$$\mathrm{Response}=\mathrm{Background}+\left\{\frac{\mathrm{Signal}-\mathrm{Background}}{1+\left({\mathrm{Kd}}^{\mathrm{Hill}\;\mathrm{Slope}}/{\mathrm{Dose}}^{\mathrm{Hill}\;\mathrm{Slope}}\right)}\right\}$$

The Hill slope was set to −1. Curves were fitted using nonlinear least squares fit with the Levenberg–Marquardt algorithm.

### 2.3. Western Blotting

For the in vitro analysis, H1299, A549, and THP-1 cells were treated with the vehicle or Q702. For assessing Axl inhibition by Q702, H1299 cells were treated with Q702 at various concentrations for 24 h and then stimulated with 200 ng/mL human Gas6 for 1 h. For Mer inhibition by Q702, A549 cells were pretreated with the indicated Q702 concentrations, treated with Pervanadate, and then stimulated with 200 ng/mL human Gas6 for 1 h. For CSF1R inhibition by Q702, THP-1 cells were treated with Q702 at various concentrations for 24 h and stimulated with 50 ng/mL human CSF1 for 5 min. After stimulation, cells were washed with ice-cold PBS and lysed with ice-cold lysis buffer to prepare cell lysates for western blotting. For the ex vivo analysis, H1299 (5 × 10^6^) or M-NFS-60 cells (1 × 10^6^) were mixed with 0.1 mL of Matrigel (50:50) and implanted into BALB/c nude mice. When the average tumor size reached ~400 mm^3^, the mice were randomized and treated with the vehicle or Q702. Tumor samples were collected 12 h after the last dose and lysed using RIPA lysis buffer. 

Whole-cell and tumor tissue protein lysates were obtained using Tris lysis buffer with protease inhibitor (Roche, Basel, Switzerland; #4693132001) and phosphatase inhibitor cocktails II and III (Sigma-Aldrich; #P5726 and #P0044). Samples were collected and kept on ice for 30 min. The supernatant was collected by centrifugation at 12,000 rpm for 10 min at 4 °C. Protein concentrations were determined using the BCA Protein Quantification Kit (Thermo Fisher Scientific, Waltham, MA, USA; Pierce, #23227). Equal volumes of protein were fractionated by SDS-PAGE, transferred to a PVDF membrane (MilliporeSigma, Burlington, MA, USA), and analyzed by probing with primary antibodies. Proteins were detected via treatment with HRP-conjugated secondary antibodies and enhanced SuperSignal^®^ Western Blot Enhancer (Thermo Fisher Scientific; #46641). The blots were read using Image Quant LAS 4000.

### 2.4. Syngeneic Tumor Mouse Models

Female BALB/c and C57BL/6 mice aged between 6 and 8 weeks were purchased from the Vital River Laboratory Animal Technology Co., Ltd. Animal experiments were conducted in accordance with the guidelines established by the Explora Biolabs Institutional Animal Care Use Committee (IACUC) of WuXi AppTec, following the guidelines of the Association for Assessment and Accreditation of Laboratory Animal Care (AAALAC). EMT6 cells were subcutaneously implanted in the right flank of each mouse. Tumor-bearing mice were randomized and treated orally every day (N = 10 per group) with the vehicle or Q702 at 30 mg/kg or the indicated doses. 

For combination studies of Q702 with the anti-PD-1 antibody, RENCA (1 × 10^6^), CT26 (1 × 10^5^), or MC38 (3 × 10^5^) cells were implanted subcutaneously in the right flank of C57BL/6 and BALB/c mice. Mice were randomized and treated with 30 mg/kg Q702 (orally every day) with or without 10 mg/kg anti-PD-1 antibody intraperitoneally twice a week starting 1 day after tumor implantation. Control mice were treated with the same dose of rat IgG2a isotype control antibody (Bio X Cell; #BE0089). Tumor volume and body weight were measured twice per week. After tumor cell inoculation, the mice were monitored daily for morbidity and mortality.

### 2.5. RNA-Sequencing

BALB/c mice were grafted with EMT6 cells. When the tumor size reached ~80 mm^3^, the mice were randomized and treated with a vehicle or 30 mg/kg Q702 orally for 7 days. On days 3, 5, and 7 (N = 3 per group), tumor samples were collected 4 h after the last dosing and snap-frozen in liquid nitrogen. RNA-sequencing profiling was conducted using the WuxiNextCODE software. The quality of all samples was validated before RNA-sequencing analysis. After raw read quality inspection with FastQC (version 0.11.2, Andrews.2010), the adapter sequences were removed from the 3′ end of the reads with a software skewer (v0.2.2). Each RNA sequence was mapped to the mouse genome (mm10) and transcriptome (GENCODE vM13) using the STAR (v2.4.2 a) software. Gene abundance was estimated using the RSEM software (v1.2.29). The library normalized factor and count per million (CPM) values were calculated using the R package edgeR (v3.8.5). Next, principal component analysis was performed to reveal the correlations among samples, and the CPM values of all genes were added by one to avoid a logarithm of zero, and were then log_2_ transformed.

### 2.6. Immune Profiling

For immunophenotyping, EMT6 tumor-bearing mice were administered the vehicle or Q702 orally for 5 days, 22 days, or as indicated. Tumor samples were collected 4 h after the last dose and homogenized using a gentleMACS dissociator (Miltenyi Biotec, North Rhine-Westphalia, Germany). Digested tissues were filtered through 70 µm cell strainers (BD Biosciences, Franklin Lakes, NJ, USA; #352350) to prepare a single-cell suspension. Cells were stained with CD45-FITC (BD Biosciences; #553080, clone 30-F11), CD45-Alexa Fluor 700 (BD Biosciences; #560510, clone 30-F11), CD3-APC-Cy7 (BD Biosciences; #560590, clone 17A2), CD4-BUV496 (BD Biosciences; #564667, clone GK1.5), CD8a-BUV737 (BD Biosciences; #564297, clone 53-6.7), CD25-BV786 (BD Biosciences; #564023, clone PC61), CD11b-Alexa Fluor 700 (BD Biosciences; #557960, clone M1/70), CD11b-BUV395 (BD Biosciences; #565976, clone M1/70), Ly6G-APC (BD Biosciences; #560599, clone 1A8), Ly6C-BV711 (BioLegend, San Diego, CA, USA; #128037, clone HK1.4), F4/80-BUV395 (BD Biosciences; #565614, clone T45-2342), MHC-II-BV605 (BD Biosciences; #563413, clone M5/114.15.2), CD206-PE (Thermo Fisher Scientific; #12-2061-82, clone MR6F3), MHC-I-PE (BioLegend; #116607), MHC-I-FITC (Thermo Fisher Scientific; #11-5998-81, clone 34-1-2S), E-cadherin-PE, and E-cadherin-Alexa Fluor 647 (BioLegend; #147308, clone DECMA-1) in the presence of purified rat anti-mouse CD16/CD32 (BD Biosciences; #553142, clone 2.4G2). Then, the cells were fixed and permeabilized using the Foxp3/Transcription Factor staining buffer set (Thermo Fisher Scientific; #00-5523-00). The cells were then stained with Foxp3-PE-Cy7 (Thermo Fisher Scientific; #25-5773-82, clone FJK-16s). Stained samples were analyzed using BD FACS LSR Fortessa flow cytometry, and all data were analyzed using FlowJo software.

### 2.7. Intracellular Staining

For intracellular cytokine staining, tumor samples or peripheral blood cells were collected 4 h after the last dose. Tumor samples were homogenized using a gentleMACS dissociator and then filtered through 70 µm cell strainers to prepare a single-cell suspension. Blood cells were lysed with a red blood cell lysis solution. Cells were stimulated with PMA + Ionomycin in the presence of brefeldin A (BD Biosciences; #555029) and monensin (#554724) for 4–6 h. The cells were stained with CD45-BV750 (BioLegend; #103157, clone 30-F11), CD3-APC-Cy7 (BioLegend; #100222, clone 17A2), CD4-Alexa Fluor 700 (BioLegend; #100430, clone GK1.5), and CD8a-Pacific Orange (Thermo Fisher Scientific; #MCD0830, clone 5H10) in the presence of purified rat anti-mouse CD16/CD32 and then fixed and permeabilized using the Foxp3/Transcription Factor staining buffer set. The cells were then stained with IFN-γ-PE (BD Biosciences; #554412, clone XMG1.2) or GranzymeB-Alexa Fluor 647 (BioLegend; #515406, clone GB11). Samples were analyzed using CYTEK Aurora spectral flow cytometry (CYTEK Biosciences, Fermont, CA, USA), and all data were analyzed using FlowJo software.

### 2.8. B16F10-OVA Model and Tetramer Staining

Female C57BL/6 mice aged between 7 and 8 weeks were purchased from Shanghai Lingchang Biotechnology Co., LTD, China. B16-OVA tumor cells (2 × 10^5^) were implanted subcutaneously (s.c.) into the right flank of mice. When tumor size reached 50 mm^3^, mice were randomized and treated intraperitoneally with the vehicle or 30 mg/kg Q702 (orally every day) with or without 10 mg/kg anti-PD-1 antibody twice a week. On day 9, tumor samples were collected and processed as single-cell suspensions. Cells were stained with CD45-BV785 (BioLegend; #103149, clone 30-F11), CD3-BUV395 (BD Biosciences; #740268, clone 17A2), CD4-BV421 (BioLegend; #100438, clone GK1.5), CD8-FITC (MBL International Corporation, Woburn, MA, USA; #D271-4, clone KT15), and SIINFEKL-H-2Kb OVA tetramer-PE (MBL International Corporation; #TB-5001-1). Stained samples were analyzed using BD FACS LSR Fortessa flow cytometry, and all data were analyzed using FlowJo software.

### 2.9. Statistical Analysis

The results of the mouse efficacy studies are shown as mean ± SEM. The significance of the difference between the control and treatment groups was compared using one-way ANOVA. The significance of the difference between the vehicle- and Q702-treated groups was analyzed using unpaired Student’s *t*-test. Statistical significance was calculated using GraphPad Prism software (GraphPad Software, La Jolla, CA, USA).

## 3. Results

### 3.1. Q702 Markedly Inhibits Axl, Mer, and CSF1R 

**Figure 1:**
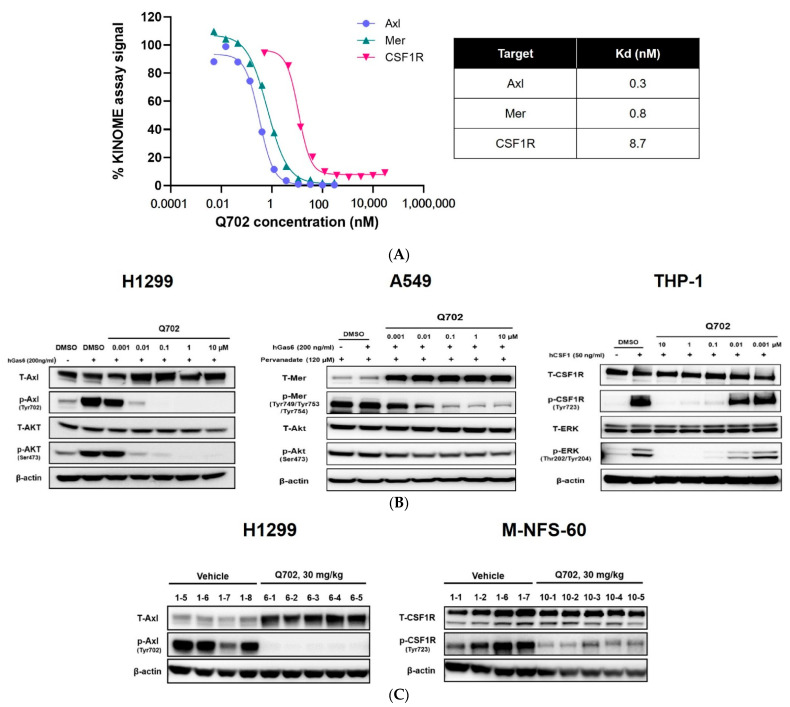
Q702 markedly inhibits the activation of Axl, Mer, and CSF1R in various in vitro and in vivo systems. (**A**) LanthaScreen equilibrium binding and biochemical binding assessment for the Kd for Axl, Mer, and CSF1R was performed. (**B**) H1299, A549, and THP-1 cells were treated with 200 ng/mL human Gas6 or 50 ng/mL human CSF1 in the presence of Q702. Cells were lysed, and the cell lysates were probed for phosphorylation of Axl (Tyr702), AKT (Ser473), Mer (Tyr749/Tyr753/Tyr754), CSF1R (Tyr723), or ERK (Thr202/Tyr204). (**C**) H1299 (5 × 10^6^) cells or M-NFS-60 (1 × 10^6^) cells were inoculated subcutaneously into the left flank of BALB/c nude mice. When the tumor size reached 200–300 mm^3^, mice were randomized and treated with vehicle or Q702 (30 mg/kg) orally once daily for one week. Tumors were lysed, and the tumor lysates were probed for phosphorylation of Axl (Tyr702) and CSF1R (Tyr723). T-Axl, total Axl; p-Axl, phosphorylated Axl; T-AKT, total AKT; p-AKT, phosphorylated AKT; T-Mer, total Mer; p-Mer, phosphorylated Mer; T-CSF1R, total CSF1R; p-CSF1R, phosphorylated CSF1R; T-ERK, total ERK; p-ERK, phosphorylated ERK. Original Western blot images can be found in Figures S3–S7.

The activity and functional potency of Q702 were assessed using a kinase-domain binding-based assay. Q702 bound to the kinase domains corresponding to Axl, Mer, and CSF1R with IC_50_ values of 0.3 nM, 0.8 nM, and 8.7 nM, respectively (Figure 1A).

Axl and Mer phosphorylation was notably enhanced by the human Gas6, a ligand of Axl and Mer; however, Gas6-induced phosphorylation was significantly inhibited by Q702 pretreatment in a concentration-dependent manner in H1299 and A549 cells. Furthermore, CSF1-induced CSF1R phosphorylation was inhibited by Q702 pretreatment in THP-1 cells (Figure 1B). These results indicate that Q702 is a consistently potent inhibitor for Axl, Mer, and CSF1R, with high binding affinity and functional potency. 

Next, we investigated Axl and CSF1R target engagement in a more physiological in vivo setting. H1299 or M-NFS-60 tumor-bearing mice were treated with Q702 for seven days, and target engagement was investigated in the tumor samples (Figure 1C). Q702 treatment inhibited the phosphorylation of Axl and CSF1R in H1299 and M-NFS-60 tumor samples, respectively, indicating that Q702 inhibits Axl and CSF1R activation in a physiological setting.

### 3.2. Q702 Exhibits Antitumor Activity by Immune Modulation

**Figure 2:**
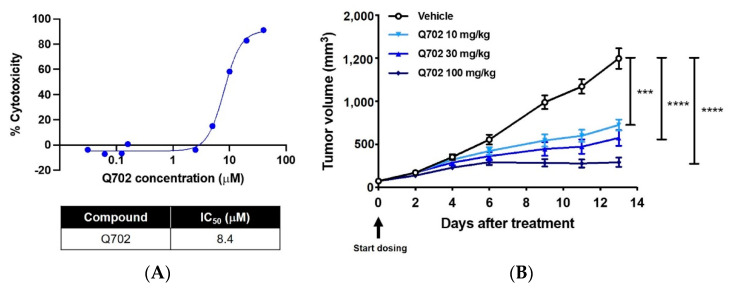
Q702 shows antitumor activity in an EMT6 syngeneic mouse model. (**A**) EMT6, mouse triple-negative breast cancer cells were treated with Q702 at the indicated concentrations for 72 h. Cell viability was measured using the CellTiter-Glo assay system. Luminescence units were normalized to those of untreated cells and are presented as the percentage of cell growth inhibition. IC_50_, the inhibition concentration of a drug where the response is reduced by half. (**B**) EMT6 (1 × 10^6^) cells were inoculated subcutaneously into the left flank of BALB/c mice. When the tumor size reached 60–80 mm^3^, mice were randomized and treated with vehicle or Q702 (10, 30, or 100 mg/kg) orally once daily for two weeks (N = 8/group). Tumor volumes were measured from the start of treatment and the results are shown as mean ± SEM. *** *p* < 0.001, **** *p* < 0.0001, one-way ANOVA.

The effect of Q702 on the growth of EMT6 cells in vitro was not potent, with an IC_50_ of 8.4 µM (Figure 2A); however, it inhibited tumor growth in immunocompetent mice in a dose-dependent manner, with 54.3%, 64.9%, and 84.6% tumor growth control achieved with 10, 30, and 100 mg/kg Q702, respectively (Figure 2B). Surprisingly, tumor growth in immunocompromised mice was not significantly affected by a higher dose of 100 mg/kg or 30 mg/kg Q702 and no dose-dependent antitumor activity of Q702 was observed, compared to the data in Figure 2B (Supplementary Figure S1A,B). Therefore, Q702 induces antitumor activity through immune modulation rather than through direct cytotoxicity to tumor cells.

**Figure 3:**
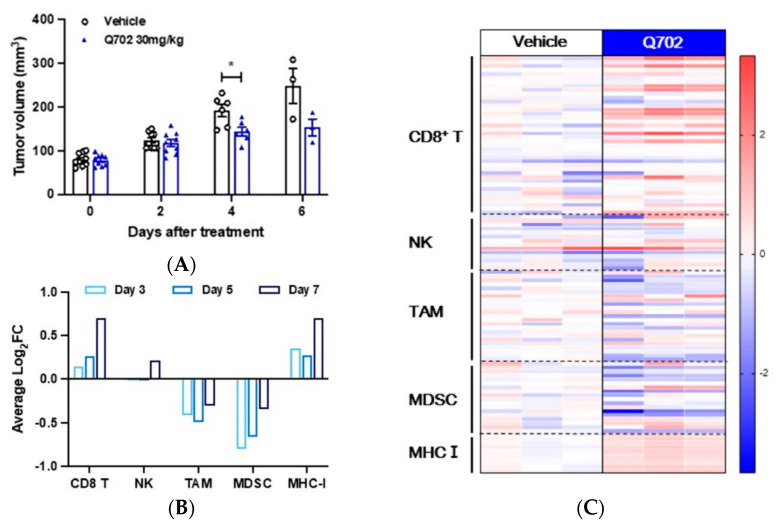
Q702 induces distinct profile changes in immune cell signature genes within the TME. EMT6 (1 × 10^6^) cells were implanted into BALB/c mice. When the tumor size reached 60–80 mm^3^, mice were randomized and treated with vehicle or Q702 (30 mg/kg) orally once daily for the indicated days. (**A**) Tumor volumes were measured on days 2, 4, and 6 after the start of treatment, and the results are shown as mean ± SEM. * *p* < 0.05, unpaired student’s *t*-test. (**B**) On days 3, 5, and 7, the tumor samples were lysed and RNA-seq analysis was performed (N = 3 mice/group). Individual transcript counts from each tumor sample were normalized with an average count per million (CPM) of the respective transcripts from the vehicle group to demonstrate gene expression changes after Q702 treatment. The immune signature gene set and MHC-I gene set were analyzed to determine the immune activation status in the tumor microenvironment. The graph shows the average log2-fold-change (log2FC) of the signature genes for each immune cell and the MHC-I gene set. (**C**) RNA-seq heatmap shows gene expression levels of general immune cell markers modulated by Q702 relative to the vehicle group. The heatmap was plotted with log2FC from the vehicle and Q702 groups for individual genes. The color scale denotes the normalized gene expression level. TAM, tumor-associated macrophages; NK, natural killer cells; MDSC, myeloid-derived suppressor cells.

To investigate the mechanism by which Q702 treatment contributes to the immune landscape within the TME, whole transcriptome RNA-seq was performed on tumor samples at 3, 5, and 7 days post-treatment with vehicle or Q702. Tumor samples were then analyzed using a signature gene set to evaluate altered signature gene sets for distinct immune cell subsets. Although no significant antitumor activity was observed in the EMT6 syngeneic mouse model after treatment with Q702 for 7 days (Figure 3A), Q702 increased the expression of CD8 T signature genes, natural killer (NK) signature genes, and major histocompatibility complex class I (MHC-I) signature genes but decreased the expression of MDSC signature genes and TAM signature genes in a time-dependent manner (Figure 3B). 

Figure 3C shows heatmaps representing the gene-level expression of individual genes that code for common immune cell markers, on day seven post-treatment with the vehicle or Q702. Consistent with the altered pattern of expression of the signature genes for each immune cell subset in Figure 3B, Q702 enriched each signature gene related to CD8 T and NK cells. Conversely, the expression of each signature gene for immunosuppressive cells, such as MDSCs and TAMs, was reduced in the TME of the Q702-treated group compared with that in the vehicle group. Interestingly, the expression of signature genes of MHC-I increased after Q702 treatment, suggesting that the susceptibility of cancer cells could be increased by Q702. Consequently, our data demonstrate that Q702 promotes antitumor immunity by preventing the immune escape of tumor cells and activating the immune system in the TME.

### 3.3. Q702 Induces Drastic Changes in the Immune Cell Population in the TME

**Figure 4:**
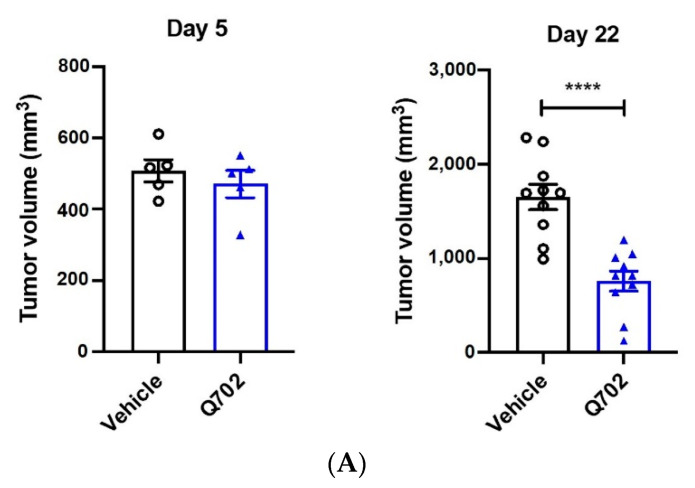

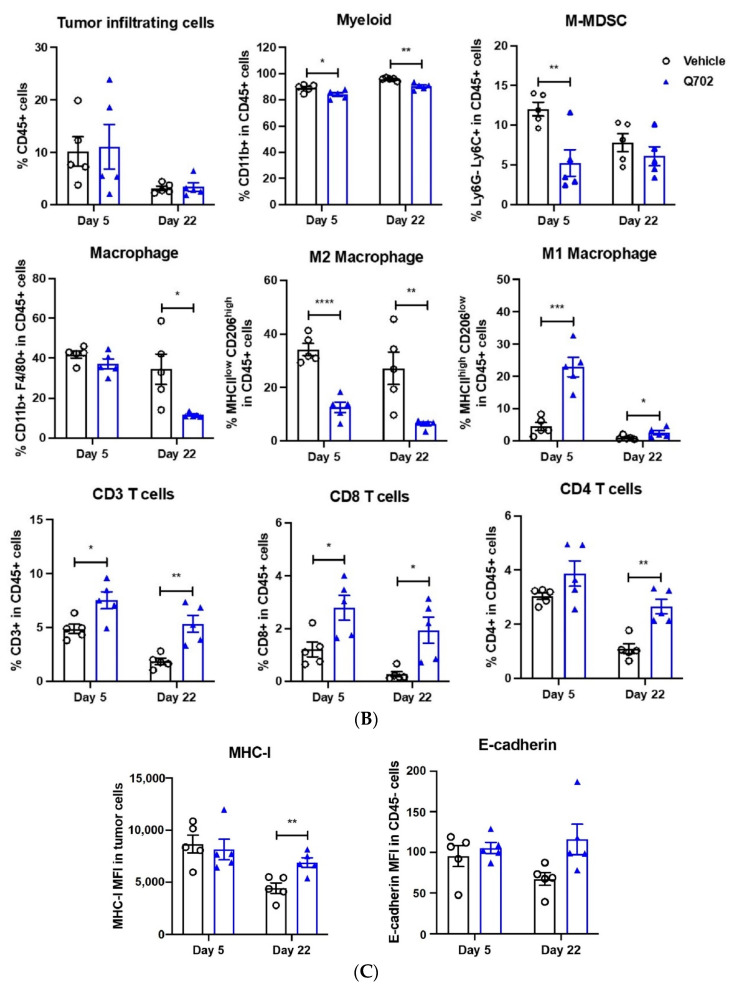
Q702 induces changes in immune cell populations in the tumor microenvironment. EMT6 tumor-bearing BALB/c mice were treated with Q702 at 30 mg/kg orally once daily. (**A**) Tumor volumes were measured 4 and 21 days after the start of treatment, and the results are shown as mean ± SEM. (**B**,**C**) Tumor samples were collected on days 5 and 22. (**B**) The graph shows the percentage of tumor-infiltrating cells (CD45^+^), myeloid cells (CD11b^+^), M-MDSCs (CD11b^+^Ly6C^+^), macrophages (CD11b^+^F4/80^+^), M1 macrophages (MHC II^high^CD206^low^), M2 macrophages (MHC II^low^CD206^high^), CD3 T cells (CD3^+^), CD8 T cells (CD3^+^CD8^+^), and CD4 T cells (CD3^+^CD4^+^) on days 5 and 22. (**C**) Graphs show MHC-I and E-cadherin expression levels in tumors (CD45-negative cells). * *p* < 0.05, ** *p* < 0.01, *** *p* < 0.001, **** *p* < 0.0001, unpaired Student’s *t*-test.

To understand the effect of Q702 on the TME over time, immune cell subpopulations were compared after 5 or 22 days of treatment with either Q702 or the vehicle in an EMT6 syngeneic breast carcinoma model. The tumor volume on day 5 was similar between the vehicle and Q702 treatment groups; however, on day 22, there was a significant decrease in the tumor volume in the Q702-treated group (Figure 4A). Major histocompatibility complex class II (MHC-II) and CD206 were used to distinguish classically (M1) and alternatively (M2) activated macrophages after total macrophages were gated with F4/80 and CD11b (Supplementary Figure S2). Q702 treatment for 5 days reduced the percentage of tumor-infiltrating CD11b^+^Ly6G^−^Ly6C^+^ monocytes (monocytic MDSCs, M-MDSCs) by 2.3-fold from 12% to 5.3%. In contrast, the percentage of CD11b^+^ F4/80^+^MHC-II^+^CD206^−^ macrophages (M1 macrophages) increased more than 5-fold, from 4% to 23%. In addition, the percentage of CD11b^+^ F4/80^+^MHC-II^−^CD206^+^ macrophages (M2 macrophages) decreased by 2.7-fold, from 34% to 13%, resulting in a decrease in the percentage of M2 macrophages persisting through day 22. As a result, the proportion of total macrophages (CD11b^+^F4/80^+^) in the TME was significantly reduced (Figure 4B).

In addition to modulating the innate immune response, Q702 can also regulate the activity of adaptive immune cells, such as T cells. On days 5 and 22, CD45^+^CD3^+^CD4^−^CD8^+^ (CD8 T cell) populations were significantly increased in the Q702-treated group compared to that in the vehicle treatment group. Overall, an increase in the proportion of adaptive immune cells, such as CD4 and CD8 T cells, and a decrease in the proportion of innate immune-suppressive cells, such as M2 macrophages and M-MDSCs, was observed in the Q702-treated group. Thus, Q702 modulates the immune cell population in the TME by changing the proportion of both innate and adaptive immune cells.

Since the Q702-treated group exhibited an increased MHC-I expression (Figure 3B,C), we evaluated whether Q702 affected the protein expression level of MHC-I in tumor cells by flow cytometry. Although the expression of signature genes of MHC-I on day 7 was higher in Q702-treated group than in those of the vehicle group (Figure 3B), the level of MHC-I protein expression in the CD45-negative cells was similar between the two groups on day 5 (Figure 4C). However, MHC-I expression in the vehicle group on day 22 decreased compared to that on day 5, indicating a tumor immune evasion mechanism by which tumor cells could escape T cell-mediated immunosurveillance. In contrast, the expression level of MHC-I in the Q702-treated group did not decrease on day 22. Due to this, the Q702-treated group maintained a higher MHC-I expression level than the vehicle group. 

Axl plays an important role in tumor cells, especially in EMT. Therefore, we investigated whether Q702 affected E-cadherin expression, a hallmark of epithelial cells in CD45-negative cells (Figure 4C). The vehicle- and Q702-treated groups showed similar E-cadherin expression levels in the CD45-negative cells on day 5; however, the E-cadherin expression levels were relatively higher in the Q702-treated group than in the vehicle group on day 22. 

Considering that there was no difference in E-cadherin expression on day 5 between the vehicle- and Q702-treated groups, these data suggest that Q702 expands the population of epithelial cells in the TME by inhibiting EMT rather than by promoting the transformation of existing mesenchymal cells to epithelial cells. In conclusion, Q702 remodels the TME toward tumor immunogenicity and triggers the activation of the innate and adaptive immune systems.

### 3.4. Q702 Increases IFN-γ Producing-NK Cells and T Cells 

Cytokines released from immune cells play a critical role in the regulation of immune responses. Cytokine levels regulate the TME, alter the proliferation and differentiation of immune cells, and influence the metastasis of cancer cells [15]. In particular, interferon gamma (IFN-γ) and granzyme B are known to increase tumor immunogenicity, inhibit tumor cell proliferation, and enhance the cytotoxic function of natural killer (NK) cells and cytotoxic T lymphocytes (CTLs) [16,17].

**Figure 5:**
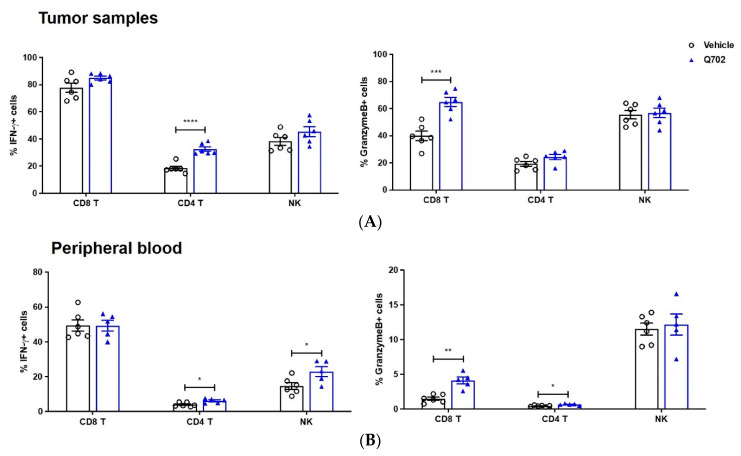
Q702 enhances effector function of T and NK cells in peripheral blood as well as in tumor samples. EMT6 (1 × 10^6^) cells were inoculated subcutaneously into the left flank of BALB/c mice. When the tumor size reached 110 mm^3^, mice were randomized and treated with vehicle or Q702 (30 mg/kg) orally once daily for 7 days. Tumor and peripheral blood samples were collected 4 h after the last dose. Cells from tumor samples (**A**) or blood cells (**B**) were stimulated with PMA + Ionomycin for 4–6 h and then stained for IFN-γ or granzyme B detection. Graphs show the percentage of IFN-γ- or granzyme B-producing CD8 T, CD4 T, or NK cells. * *p* < 0.05, ** *p* < 0.01, *** *p* < 0.001, **** *p* < 0.0001, unpaired Student’s *t*-test. NK, natural killer cells.

Therefore, we also evaluated the effects of Q702 on key moderators of cell-mediated immunity, including IFN-γ and granzyme B. When T cells or NK cells from tumor samples were stimulated in vitro with phorbol 12-myristate 13-acetate (PMA) and ionomycin, the proportion of IFN-γ-producing CD4 T cells significantly increased in the Q702 treatment group. Interestingly, the proportion of granzyme B^+^ CD8 T cells was significantly higher in the Q702-treated group; however, the percentage of granzyme B^+^ CD4 T cells or NK cells did not differ between the vehicle and Q702 treatment groups (Figure 5A). T cells or NK cells in the peripheral blood showed a pattern similar to that of tumor samples, indicating that Q702 treatment increased the proportion of IFN-γ-producing CD4 T and NK cells and granzyme B^+^ CD8 T cells (Figure 5B). Collectively, these data indicate that cytokine profiling of peripheral blood can provide a systematic view of the immune status of Q702-treated tumors because Q702 enhances the effector function of T and NK cells by increasing cytokine levels in both tumor samples and peripheral blood.

### 3.5. Combination of Q702 and Anti-PD-1 Antibody Exhibits Good Antitumor Activity

**Figure 6:**
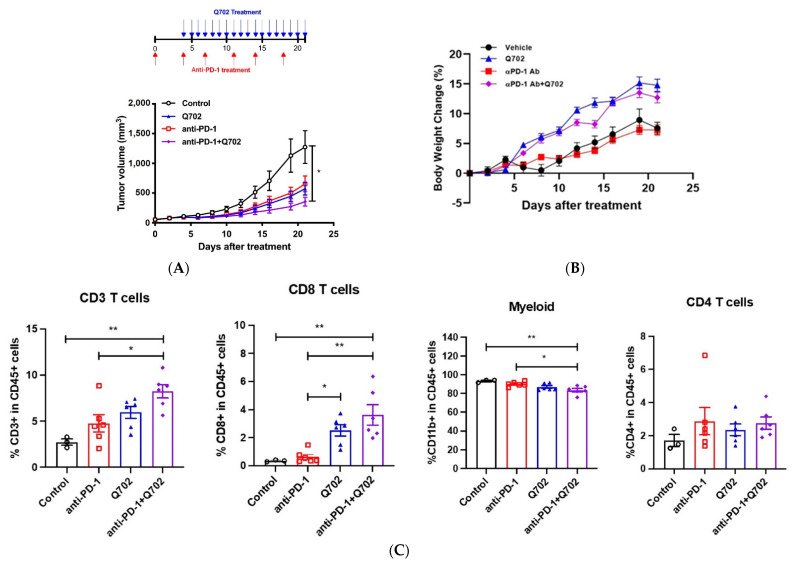

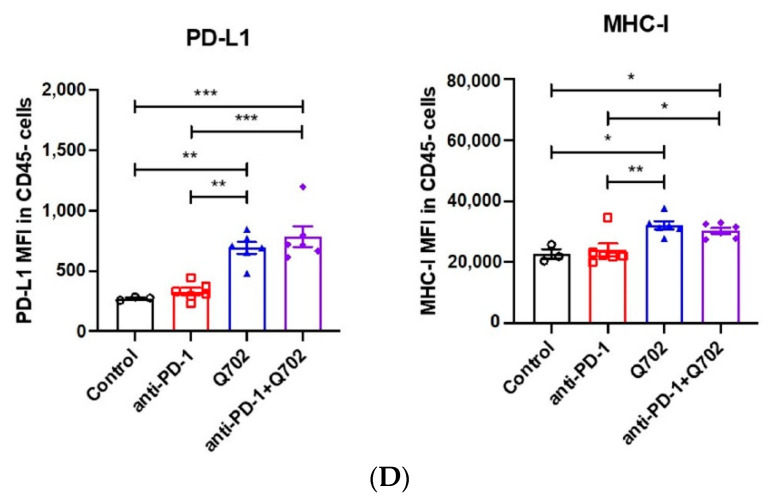
Combined treatment with Q702 and anti-PD-1 antibody inhibits EMT6 tumor growth in vivo. EMT6 (1 × 10^6^) cells were implanted into the left flank of BALB/c mice. When the tumor size reached 55 mm^3^, mice were randomized and treated with vehicle or anti-PD-1 antibody (10 mg/kg) intraperitoneally twice a week. Four days later, mice were treated with either Q702 (30 mg/kg) orally once daily or with a combination of Q702 and anti-PD-1 antibody. (**A**) Dose schedule and tumor growth in vivo are shown. (**B**) Graph shows changes in mouse body weight over time after treatment. (**C**,**D**) Tumor samples were collected on day 21 post-dosing and immune profiling was performed. (**C**) Graphs show the percentages of tumor-infiltrating CD3 T (CD3^+^), CD8 T (CD3^+^CD8^+^), CD4 T (CD3^+^CD4^+^), and myeloid (CD11b^+^) cells. (**D**) Graphs show PD-L1 and MHC-I expression levels in tumors (CD45-negative cells). * *p* < 0.05, ** *p* < 0.01, *** *p* < 0.001, one-way ANOVA.

We observed that Q702 induces tumor growth inhibition by enhancing the accumulation of infiltrated CD8 T cells, increasing MHC-I expression, and inhibiting EMT in tumor cells. Since insufficient CD8 T cells and tumor immunogenicity in the TME are common causes of the limited efficacy of anti-PD-1 antibodies, these results suggest the potential of Q702 as a promising agent when combined with anti-PD-1 antibody therapy to overcome this limitation. Therefore, we evaluated the antitumor activity of Q702 in combination with an anti-PD-1 antibody in an EMT6 syngeneic mouse model. Q702 or anti-PD-1 antibody monotherapy showed partial antitumor activity in the EMT6 model, whereas the combination of Q702 and anti-PD-1 antibody significantly enhanced tumor growth inhibition (TGI) activity without body weight loss (Figure 6A,B).

To determine how the combination treatment increased TGI, flow cytometry analysis was performed on whole tumors after 21 days of Q702 treatment with or without the anti-PD-1 antibody. The infiltrated immune cell population did not differ significantly between the anti-PD-1 antibody treatment group and the vehicle treatment group. However, Q702 increased the infiltration of CD8 T cells and decreased the accumulation of the myeloid cell population (Figure 6C). In addition, Q702 increased MHC-I and PD-L1 expression in CD45-negative cells in the TME compared to that in the vehicle group (Figure 6D). These results suggest that Q702 not only enhances the antitumor efficacy of anti-PD-1 antibody but can also overcome the limited efficacy of anti-PD-1 by enhancing tumor immunogenicity and inducing CD8 T cell infiltration into the TME.

### 3.6. Q702 Expands the Tumor Antigen-Specific CD8 T Cell Population in the TME

**Figure 7:**
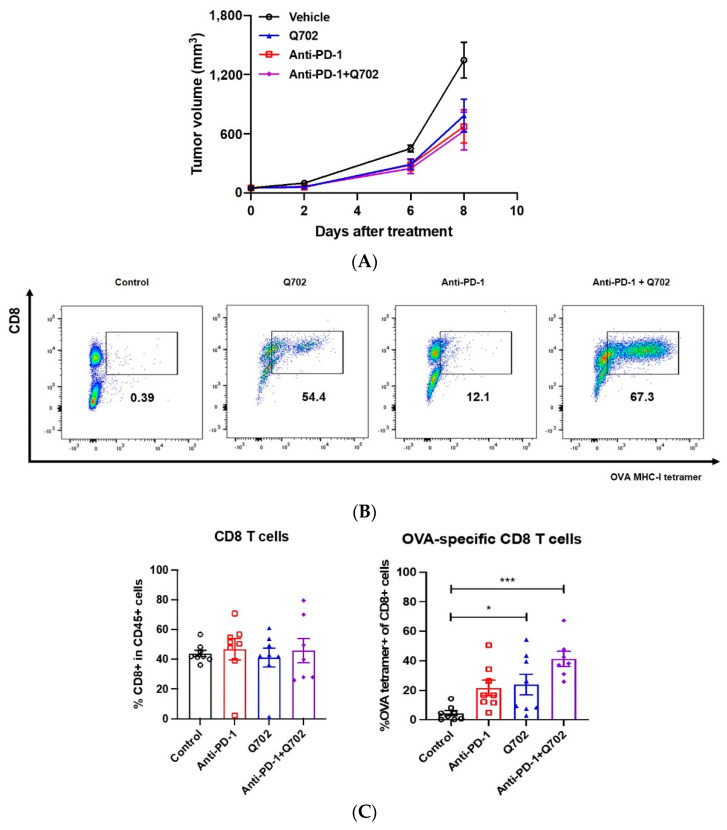
Q702 enhances the infiltration of tumor antigen-specific CD8 T cells into tumors. B16F10-OVA (2 × 10^5^) cells were inoculated subcutaneously into the left flanks of female C57BL/6 mice. When the tumor size reached approximately 50 mm^3^, mice were randomized and treated with vehicle, anti-PD-1 antibody (10 mg/kg, twice a week, intraperitoneally), Q702 (30 mg/kg, once daily, oral administration), or with a combination of anti-PD-1 and Q702 for 9 days. (**A**) The graph shows the tumor growth in vivo (mean ± SEM). (**B**,**C**) Tumor samples were collected on day 9 post-treatment and OVA-specific CD8 T cells from tumor samples were quantified by SIINFEKL-tetramer staining. (**B**) Representative scatter plots show OVA-MHC-I-tetramer staining. (**C**) Graphs show the average percentages of CD8 T cells and OVA-specific CD8 T cells in tumor samples. * *p* < 0.05, *** *p* < 0.001, one-way ANOVA.

To determine whether the cells involved in Q702-induced TGI treatment were class I MHC molecule-restricted and epitope-specific conventional CD8 T cells, and to evaluate the change in the epitope-specific conventional CD8 T cell population induced by the combination of Q702 and anti-PD-1 antibody, we performed experiments using a syngeneic model with B16F10-ovalbumin (OVA) melanoma cells. Q702 or anti-PD-1 antibody monotherapy induced partial antitumor activity in the B16F10 model, and there was no difference in TGI between the combination treatment group and the Q702 or anti-PD-1 antibody monotherapy groups (Figure 7A). However, Q702 treatment significantly induced the accumulation of H-2Kb/SIINFEKL tetramer-positive CD8 T cells in the TME, which recognize the tumor antigen OVA on H-2Kb class I MHC molecules. Interestingly, although there was no difference in the efficacy and CD8 T cell infiltration between the monotherapy and combination therapy groups (Figure 7B), the number of tumor antigen-specific CD8 T cells in the combination group increased compared to that in the Q702 or anti-PD-1 antibody monotherapy groups (Figure 7C). In conclusion, these data support the hypothesis that Q702 acts cooperatively with checkpoint inhibitors, suggesting that Q702 is an effective treatment agent for overcoming resistance to current cancer immunotherapy involving T cell checkpoint inhibitors.

### 3.7. Combination of Q702 and Anti-PD-1 Antibody Enhances Antitumor Activity in Renal or Colon Cancer Syngeneic Models

**Figure 8:**
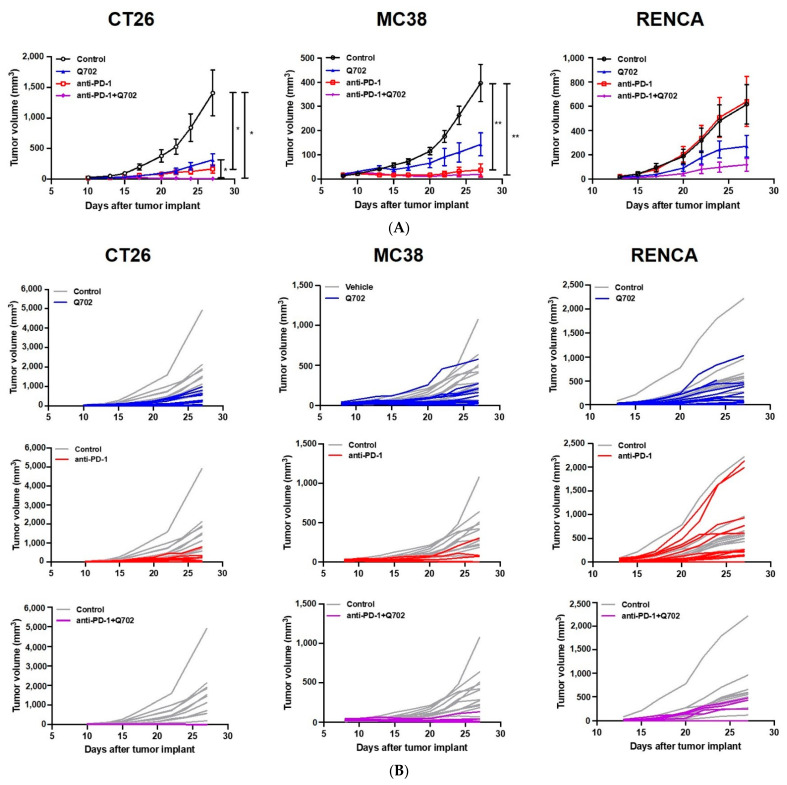
Combination treatment with Q702 and anti-PD-1 antibody enhances tumor growth inhibition in various syngeneic mouse tumor models. (**A**) RENCA mouse renal adenocarcinoma cells (1 × 10^6^) were inoculated subcutaneously into BALB/c mice. CT26 mouse colon carcinoma cells (1 × 10^5^) were implanted into BALB/c mice. MC38 mouse colon adenocarcinoma cells (3 × 10^5^) were inoculated subcutaneously into C57BL/6 mice. Mice (N = 10/group) were randomized one day after tumor implantation and treated with either vehicle, anti-PD-1 (10 mg/kg, intraperitoneally twice a week), Q702 (30 mg/kg, orally once daily), or a combination of anti-PD-1 antibody and Q702. Tumor growth inhibition data are presented as mean ± SEM. * *p* < 0.05, ** *p* < 0.01, one-way ANOVA. (**B**) Graphs show the tumor growth in individual mice in each treatment group.

Encouraged by the advantages of combination therapy observed in the EMT6 syngeneic model, we next evaluated the antitumor activity of Q702 in combination with an anti-PD-1 antibody in various syngeneic mouse models, such as CT26, MC38, and RENCA syngeneic models (Figure 8). In the CT26 syngeneic colon cancer model, Q702 or anti-PD-1 antibody monotherapy group showed a similar TGI in mice (77% and 88% at day 27, respectively), but TGI was significantly enhanced when administered in combination (99.7%). Similarly, in the MC38 syngeneic colon cancer model, Q702 or anti-PD-1 monotherapy did not show significant difference in TGI (64% and 91% on day 27, respectively), whereas the combination treatment significantly improved TGI (95%). In addition, in the CT26 and MC38 models, the combination therapy showed a synergetic effect on antitumor efficacy compared to the anti-PD-1 monotherapy, as more than 50% of mice showed a complete response to the therapy. More interestingly, in the RENCA renal adenocarcinoma model, the combination of Q702 with anti-PD-1 antibody remarkably enhanced TGI (81%), despite no reduction in tumor growth by the anti-PD-1 antibody alone. Collectively, our results indicate that the combination of Q702 with the anti-PD-1 antibody potentiates antitumor activity against not only anti-PD-1-sensitive tumors but also against anti-PD-1-resistant tumors.

## 4. Discussion

Currently, most cancer immunotherapy reagents focus on T cells because the therapeutic reactivation of exhausted and tumor-infiltrating T cells by immune checkpoint blockade (ICB) provides unprecedented clinical benefits to patients with cancer. However, reactivation of T cells through ICBs such as anti-PD-1 or anti-CTLA4 antibodies alone cannot antagonize all immune resistance mechanisms, and a significant proportion of patients with cancer do not respond to ICB treatment [19,20]. Considering that innate immunity plays an important role in stimulating and supporting adaptive immunity, activating innate immunity to reactivate exhausted T cells in the TME can be an effective treatment strategy to enhance both innate and adaptive immunity. Therefore, novel targets that are not limited to T cells have been explored in several clinical trials worldwide. In this study, we identified Q702, a molecule that simultaneously inhibits Axl, Mer, and CSF1R. Q702 inhibited the phosphorylation of Axl, Mer, and CSF1R in tumor samples from human tumor xenograft models as well as in Axl, Mer, or CSF1R-overexpressing cell lines. Q702 treatment induced antitumor activity and improved the efficacy of anti-PD-1 therapy by reducing the number of M2 macrophages and MDSCs, inducing M1 macrophages and cytotoxic CD8 T cells, and increasing the expression of MHC-I and E-cadherin in tumor cells in the TME.

TAMs and MDSCs exert their pro-tumorigenic effects by suppressing T cell function and promoting tumor angiogenesis, proliferation, survival, and metastasis. TAMs, the major components of non-tumor stromal cells in the TME, are important cells that secrete chemokines, cytokines, and growth factors that promote tumor development and progression, thereby creating an immunosuppressive microenvironment. Interestingly, TAMs are recruited and programmed through crosstalk with tumors. Previous studies have primarily focused on macrophage depletion strategies; however, it has recently become clear that reprogramming TAMs can be a more effective strategy. Therefore, current therapeutic strategies focus on reducing macrophage infiltration in tumor tissues and inducing the repolarization of TAMs to M1-like phenotypes to kill tumors [21]. Recent evidence has shown that anti-Axl antibody treatment increases the number of M1 macrophages [22]. In addition, neutralizing antibodies against Gas6 or ProS, ligands for Axl and Mer, increase the expression of genes encoding proinflammatory proteins related to M1 macrophages [23]. CSF1 participates in the recruitment of TAMs to tumor tissues and in the M2 polarization of TAMs. Thus, CSF1R inhibition reduces M2 macrophage marker expression and impairs the tumor-promoting activity of TAMs in tumors [24]; however, clinical studies have shown that the use of CSF1R inhibitors exerted very limited antitumor effects in patients. This is because CSF1R inhibitors block the recruitment of TAMs while simultaneously increasing polymorphonuclear MDSC (PMN-MDSC) infiltration into the TME. 

MDSCs are involved in immune suppression in the TME via various mechanisms. MDSCs inhibit the activity of T cells, NK cells, and macrophages [25] to promote tumor development and growth and immune resistance to ICB therapies by recruiting regulatory T cells [26] and expressing immunosuppressive mediators [27]. Several reports have shown that MDSCs drastically upregulated the expression of Axl, Mer, and their ligands Gas6 and ProS, whereas MDSCs from Axl- or Mer-knockout mice showed reduced suppression of T cell activity [28,29,30]. Similarly, we also observed a marked reduction in the number of MDSCs in tumors from Q702-treated mice, suggesting that Q702 can reverse the MDSC-mediated immune-suppressive environment in tumors.

Findings from a recent meta-analysis of 442 patients with various solid tumors demonstrated that MDSCs are significantly associated with poor overall survival and progression-free survival [31]. Moreover, a study has recently reported an association between a lower number of MDSCs and positive clinical responses to ICB therapies, including anti-CTLA4 and anti-PD-1 antibodies [32]. Thus, preventing the accumulation of MDSCs in the TME could potentially reduce immune suppression in tumors and enhance antitumor activity. Our results suggested that Axl and Mer inhibition could enhance T cell-centric therapies by helping the differentiation of MDSCs into macrophages and DCs. Therefore, Axl and Mer are attractive targets for reducing MDSC accumulation in the TME, and Q702 can modulate MDSC-mediated immune suppression in tumors.

Interestingly, the efficacy of Q702 treatment depends not only on immune system modulation but also on tumor cell regulation within the TME. Q702 directly affects tumor cells by regulating EMT, a crucial step that cancer cells must go through to evade the immune system. Although extensive molecular changes have been observed during EMT, a key feature is the loss of E-cadherin, which interacts with receptors on several immune cell types, particularly DCs, effector CD8 T cells, and CD4 T cells. Thus, the loss of E-cadherin inhibits immune surveillance functions such as antigen cross-presentation to CD8 T cells [33,34]. In addition, EMT can promote the immunosuppressive TME by recruiting MDSCs and M2 macrophages and may reduce susceptibility to cytotoxic T cell-mediated lysis by reducing vulnerability to apoptosis in the quasi-mesenchymal cell state [35]. Mesenchymal cell lines and the tumors formed from them express significantly lower levels of MHC-I than epithelial cell lines and their corresponding tumors. EMT-induced low MHC-I expression renders the cells vulnerable to antigen presentation and consequently leads to the attenuation of T cell-mediated lysis [10]. In our study, Q702 treatment increased E-cadherin and MHC-I expression in CD45-negative cells, indicating that Q702 has better efficacy than compounds that can modulate immune cells only and would be ideal in combination with compounds inducing ICB. Our data suggest the advantages in investigating combination of Q702 with other immune checkpoint inhibitors. In addition, Q702 induced significant TGI in an anti-PD-1 antibody-resistant RENCA syngeneic model and better TGI in combination with Q702 and anti-PD-1 antibody in various syngeneic models. These data indicate that Q702 can restore and enhance immune functions by affecting innate and adaptive immunity. Moreover, Q702 modulates the crosstalk between immune cells and tumor cells, supporting the clinical evaluation of Q702 as an immunomodulatory agent to treat patients with various cancers.

## 5. Conclusions

Axl, Mer and CSF1R inhibition by Q702 remodels the TME towards immune stimulation. In terms of immune cells in the TME, Q702 expanded M1 macrophage and CD8 T cell populations and decreased M2 macrophage and MDSC populations in the TME. Q702 also increased immunogenicity through increased expression of MHC class I and E-cadherin in tumor cells.

## Data Availability

The data presented in this study are available in the article and the Supplementary Materials.

## Supplementary Materials

The following supporting information can be downloaded at: https://www.mdpi.com/article/10.3390/cancers14194821/s1, Figure S1: Tumor growth in immunodeficient mice is not significantly affected by Q702; Figure S2: Flow cytometry gating strategy; Figure S3: Full western blots of Figure 1B, left (H1299). Figure S4: Full western blots of Figure 1B, middle (A549). Figure S5: Full western blots of Figure 1B, right (THP-1). Figure S6: Full western blots of Figure 1C, left (H1299 tumor samples). Figure S7: Full western blots of Figure 1C, right (M-NFS-60 tumor samples).

## References

[1] Zuazo M., Gato-Cañas M., Llorente N., Ibañez-Vea M., Arasanz H., Kochan G., Escors D. (2017). Molecular mechanisms of programmed cell death-1 dependent T cell suppression: Relevance for immunotherapy. Ann. Transl. Med..

[2] Beatty G.L., Gladney W.L. (2015). Gladney. Immune escape mechanisms as a guide for cancer immunotherapy. Clin. Cancer Res..

[3] Akinleye A., Rasool Z. (2019). Immune checkpoint inhibitors of PD-L1 as cancer therapeutics. J. Hematol. Oncol..

[4] Nowicki T.S., Hu-Lieskovan S., Ribas A. (2018). Mechanisms of Resistance to PD-1 and PD-L1 Blockade. Cancer J..

[5] Harlé G., Nel J., Corbier C., Touche N., Grandemange S. (2021). Tumor-associated macrophages: Shifting bad prognosis to improved efficacy in cancer therapies?. Int. J. Immunother. Cancer Res..

[6] Lei Q., Wang D., Sun K., Wang L., Zhang Y. (2020). Resistance Mechanisms of Anti-PD1/PDL1 Therapy in Solid Tumors. Front. Cell Dev. Biol..

[7] Graham D., DeRyckere D., Davies K., Earp H.S. (2014). The TAM family: Phosphatidylserine-sensing receptor tyrosine kinases gone awry in cancer. Nat. Rev. Cancer..

[8] Cheng P., Phillips E., Kim S.H., Taylor D., Hielscher T., Puccio L., Hjelmeland A.B., Lichter P., Nakano I., Goidts V. (2015). Kinome-wide shRNA screen identifies the receptor tyrosine kinase AXL as a key regulator for mesenchymal glioblastoma stem-like cells. Stem Cell Rep..

[9] Schoumacher M., Burbridge M. (2017). Key roles of AXL and MER receptor tyrosine kinases in resistance to multiple anticancer therapies. Curr. Oncol. Rep..

[10] De Matteis S., Canale M., Verlicchi A., Bronte G., Delmonte A., Crinò L., Martinelli G., Ulivi P. (2019). Advances in molecular mechanisms and immunotherapy involving the immune cell-promoted epithelial-to-mesenchymal transition in lung cancer. J. Oncol..

[11] Myers K.V., Amend S.R., Pienta K.J. (2019). Targeting Tyro3, Axl and MerTK (TAM receptors): Implications for macrophages in the tumor microenvironment. Mol. Cancer.

[12] Zhou Y., Fei M., Zhang G., Liang W.C., Lin W., Wu Y., Piskol R., Ridgway J., McNamara E., Huang H. (2020). Blockade of the phagocytic receptor MerTK on tumor-associated macrophages enhances P2 × 7R-dependent STING activation by tumor-derived cGAMP. Immunity.

[13] Werfel T.A., Cook R.S. (2018). Efferocytosis in the tumor microenvironment. Semin. Immunopathol..

[14] Lartigue J. Interest Builds in CSF1R for Targeting Tumor Microenvironment. 2018, OncLive. https://www.onclive.com/view/interest-builds-in-csf1r-for-targeting-tumor-microenvironment.

[15] Cannarile M.A., Weisser M., Jacob W., Jegg A.M., Ries C.H., Rüttinger D. (2017). Colony-stimulating factor 1 receptor (CSF1R) inhibitors in cancer therapy. J. Immunother. Cancer.

[16] Kumar V., Donthireddy L., Marvel D., Condamine T., Wang F., Lavilla-Alonso S., Hashimoto A., Vonteddu P., Behera R., Goins M.A. (2017). Cancer-associated fibroblasts neutralize the antitumor effect of CSF1 receptor blockade by inducing PMN-MDSC infiltration of tumors. Cancer Cell.

[17] Zhu Y., Knolhoff B.L., Meyer M.A., Nywening T.M., West B.L., Luo J., Wang-Gillam A., Goedegebuure S.P., Linehan D.C., DeNardo D.G. (2014). CSF1/CSF1R blockade reprograms tumor-infiltrating macrophages and improves response to T-cell checkpoint immunotherapy in pancreatic cancer models. Cancer Res..

[18] Rovida E., Sbarba P.D. (2015). Colony-stimulating factor-1 receptor in the polarization of macrophages: A target for turning bad to good ones?. J. Clin. Cell. Immunol..

[19] Arasanz H., Gato-Cañas M., Zuazo M., Ibañez-Vea M., Breckpot K., Kochan G., Escors D. (2017). PD1 signal transduction pathways in T cells. Oncotarget.

[20] Sharma P., Hu-Lieskovan S., Wargo J.A., Ribas A. (2017). Primary, adaptive, and acquired resistance to cancer immunotherapy. Cell.

[21] Ge Z., Ding S. (2020). The crosstalk between tumor-associated macrophages (TAMs) and tumor cells and the corresponding targeted therapy. Front. Oncol..

[22] Shibata T., Habiel D.M., Coelho A.L., Hogaboam C.M. (2014). Axl receptor blockade protects from invasive pulmonary aspergillosis in mice. J. Immunol..

[23] Deng T., Zhang Y., Chen Q., Yan K., Han D. (2012). Toll-like receptor-mediated inhibition of Gas6 and ProS expression facilitates inflammatory cytokine production in mouse macrophages. Immunology.

[24] Pyonteck S.M., Akkari L., Schuhmacher A.J., Bowman R.L., Sevenich L., Quail D.F., Olson O.C., Quick M.L., Huse J.T., Teijeiro V. (2013). CSF-1R inhibition alters macrophage polarization and blocks glioma progression. Nat. Med..

[25] Rashid M.H., Borin T.F., Ara R., Piranlioglu R., Achyut B.R., Korkaya H., Liu Y., Arbab A.S. (2021). Critical immunosuppressive effects of MDSC-derived exosomes in the tumor microenvironment. Oncol. Rep..

[26] Pan P.Y., Ma G., Weber K.J., Ozao-Choy J., Wang G., Yin B., Divino C.M., Chen S.H. (2010). The immunostimulatory receptor CD40 is required for T-cell suppression and T regulatory cell activation mediated by myeloid-derived suppressor cells in cancer. Cancer Res..

[27] Huang B., Pan P.Y., Li Q., Sato A.I., Levy D.E., Bromberg J., Divino C.M., Chen S.H. (2006). Gr-1^+^CD115^+^ immature myeloid suppressor cells mediate the development of tumor-induced T regulatory cells and T-cell anergy in tumor-bearing host. Cancer Res..

[28] Linger R.M., Keating A.K., Earp H.S., Graham D.K. (2010). Taking aim at Mer and Axl receptor tyrosine kinases as novel therapeutic targets for solid tumors. Expert Opin. Ther. Targets.

[29] Son H.Y., Jeong H.K. (2021). Immune Evasion Mechanism and AXL. Front. Oncol..

[30] Holtzhausen A., Harris W., Ubil E., Hunter D.M., Zhao J., Zhang Y., Zhang D., Liu Q., Wang X., Graham D.K. (2019). TAM family receptor kinase inhibition reverses MDSC-mediated suppression and augments anti-PD-1 therapy in melanoma. Cancer Immunol. Res..

[31] Gabrilovich D.I. (2017). Myeloid-derived suppressor cells. Cancer Immunol. Res..

[32] Li Y., Liu J., Gao L., Liu Y., Meng F., Li X., Qin F.X. (2020). Targeting the tumor microenvironment to overcome immune checkpoint blockade therapy resistance. Immunol. Lett..

[33] Shields B.D., Koss B., Taylor E.M., Storey A.J., West K.L., Byrum S.D., Mackintosh S.G., Edmondson R., Mahmoud F., Shalin S.C. (2019). Loss of E-cadherin inhibits CD103 antitumor activity and reduces checkpoint blockade responsiveness in melanoma. Cancer Res..

[34] Sommariva M., Gagliano N. (2020). E-cadherin in pancreatic ductal adenocarcinoma: A multifaceted actor during EMT. Cells.

[35] Zhang Y., Weinberg R.A. (2018). Epithelial-to-mesenchymal transition in cancer: Complexity and opportunities. Front. Med..

