# Supplementary material for: A Novel Selective Axl/Mer/CSF1R Kinase Inhibitor as a Cancer Immunotherapeutic Agent Targeting Both Immune and Tumor Cells in the Tumor Microenvironment
Source: Cancers (Basel). 2022 Oct 2;14(19):4821. doi: 10.3390/cancers14194821 (PMC9563311; doi:10.3390/cancers14194821)
Supplement: Supplementary file 1 [file cancers-14-04821-s001.zip › cancers-1924957-supplementary.pdf]

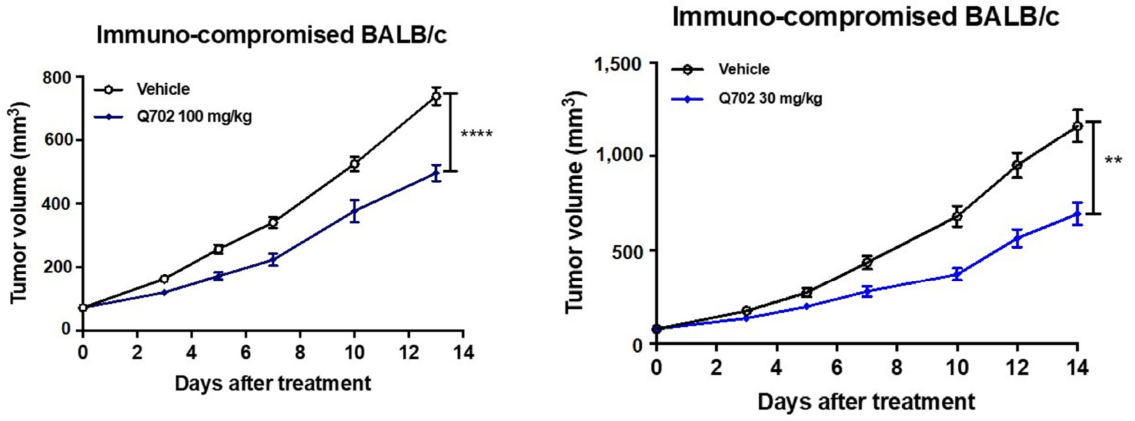

(A) (B)

Supplementary Figure S1. Tumor growth in immunodeficient mice is not significantly affected by Q702. (**A**) EMT6 (1×10^6) cells were inoculated subcutaneously into the left flank of BALB/c nude mice. When the tumor size reached 72 mm^3, mice were randomized and treated with vehicle or Q702 (100 mg/kg) orally once daily. (**B**) EMT6 (1×10^6) cells were implanted into BALB/c nude mice. When the tumor size reached 69 mm^3, mice were randomized and treated with vehicle or Q702 (30 mg/kg) orally once daily. Data are presented as mean ± SEM. ** $P < 0.01$, **** $P < 0.0001$, one−way ANOVA.

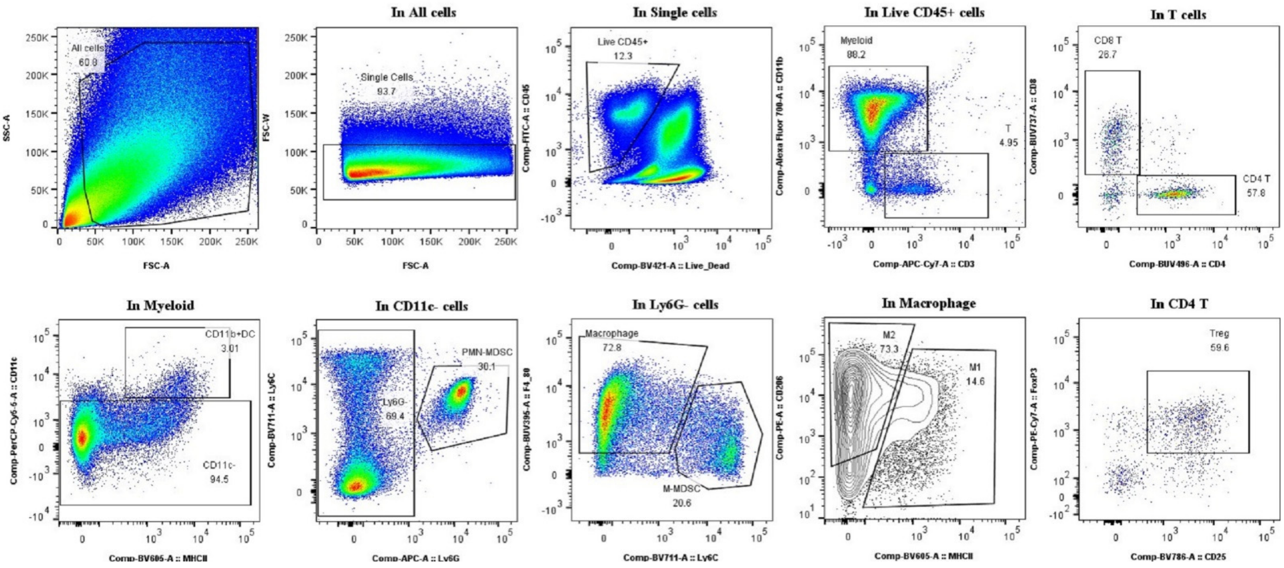

Supplementary Figure S2. Flow cytometry gating strategy. Selection of cells without debris was identified by plotting forward scatter−area and side scatter-area; single cells are separated by forward scatter-area against forward scatter−width; Dead cells were excluded with Live Dead staining and CD45$^+$ cells were selected; CD3 and CD11b were plotted to select CD3 T cells (CD45$^+$CD3$^+$CD11b$^-$) and myeloid cells (CD45$^+$CD3$^-$CD11b$^+$); CD4 T (CD3$^+$CD4$^+$CD8$^-$) and CD8 T (CD3$^+$CD4$^-$CD8$^+$) cells were identified by CD4 and CD8 staining; T regulatory cells (CD3$^+$CD4$^+$CD25$^+$Foxp3$^+$) were identified in CD4$^+$ cells by plotting Foxp3 against CD25; CD11b$^+$ DCs (CD11b$^+$CD11c$^+$MHC−II$^+$) were selected by plotting CD11c and MHC−II; PMN−MDSCs (CD11b$^+$CD11c$^-$Ly6G$^+$Ly6C$^+$) were identified by Ly6G and Ly6C; M-MDSCs (CD11b$^+$CD11c$^-$Ly6G$^-$Ly6C$^+$) and macrophages (CD11b$^+$CD11c$^-$Ly6G$^-$F4/80$^+$) were identified by plotting Ly6C and F4/80; M1 macrophages (CD11b$^+$CD11c$^-$F4/80$^+$MHC−II$^+$CD206$^-$) and M2 macrophages (CD11b$^+$CD11c$^-$F4/80$^+$MHC−II$^-$CD206$^+$) were identified by CD206 and MHC−II staining.

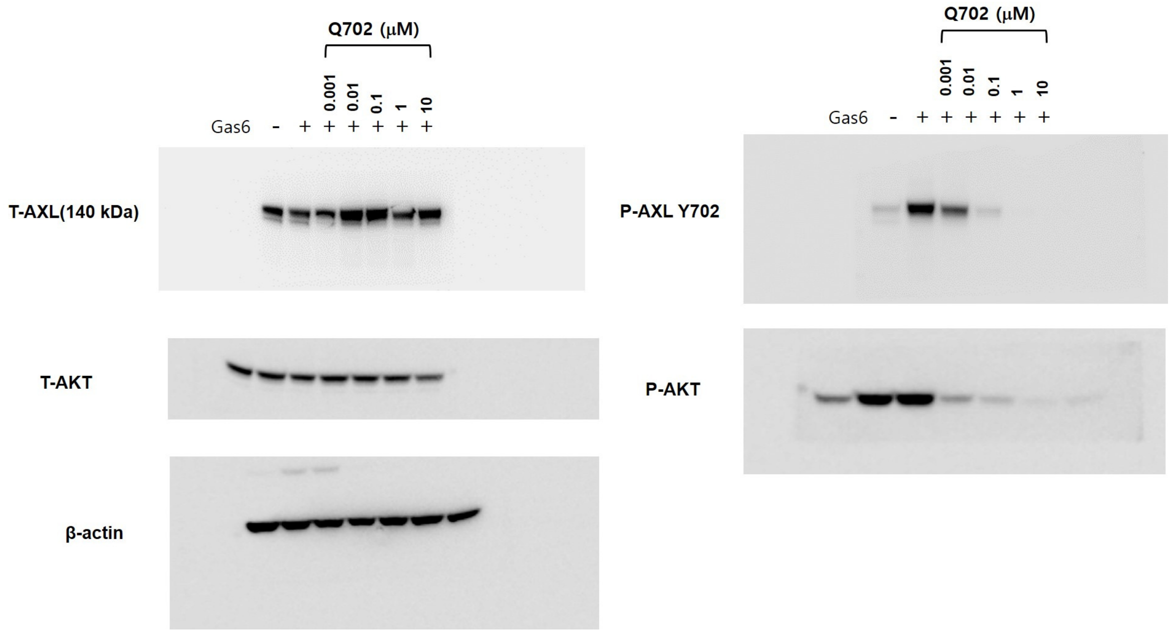

Supplementary Figure S3. Full western blots of Figure 1B, left (H1299).

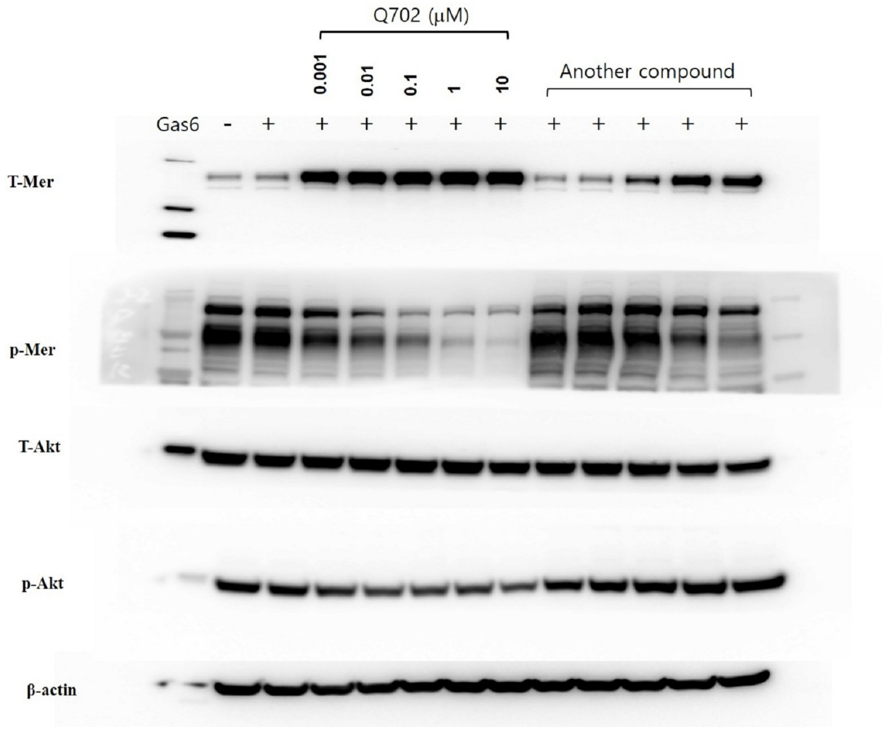

Supplementary Figure S4. Full western blots of Figure 1B, middle (A549).

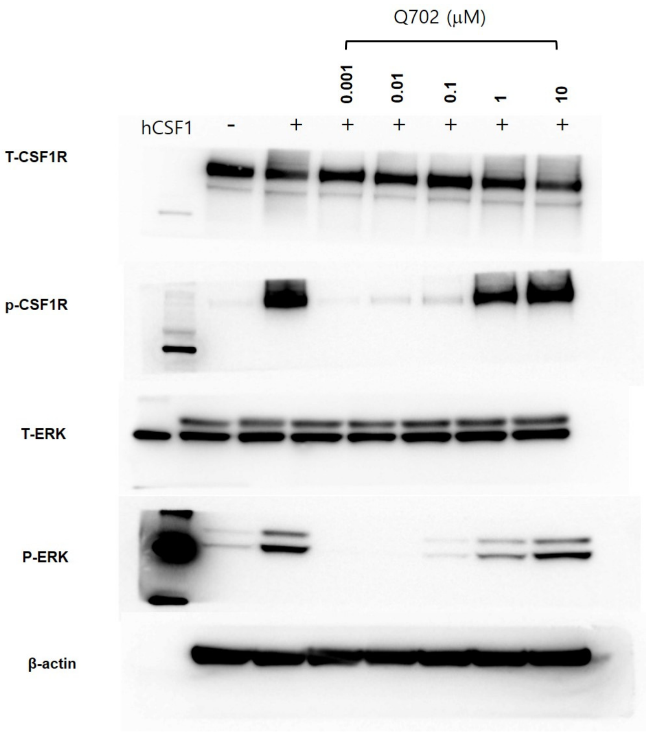

Supplementary Figure S5. Full western blots of Figure 1B, right (THP-1).

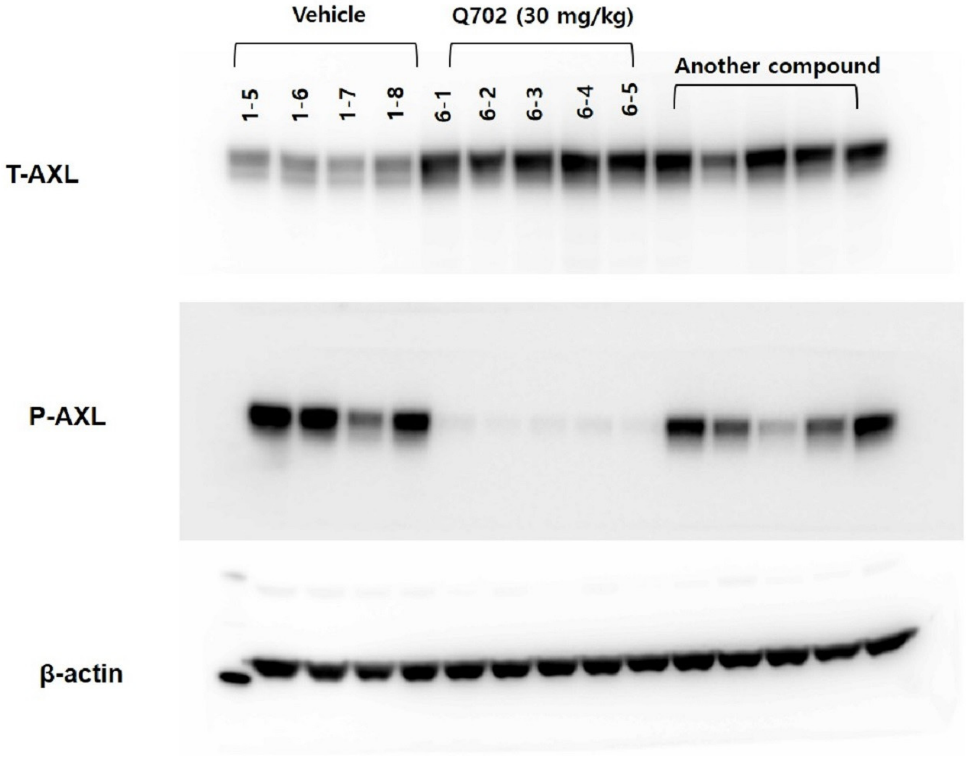

Supplementary Figure S6. Full western blots of Figure 1C, left (H1299 tumor samples).

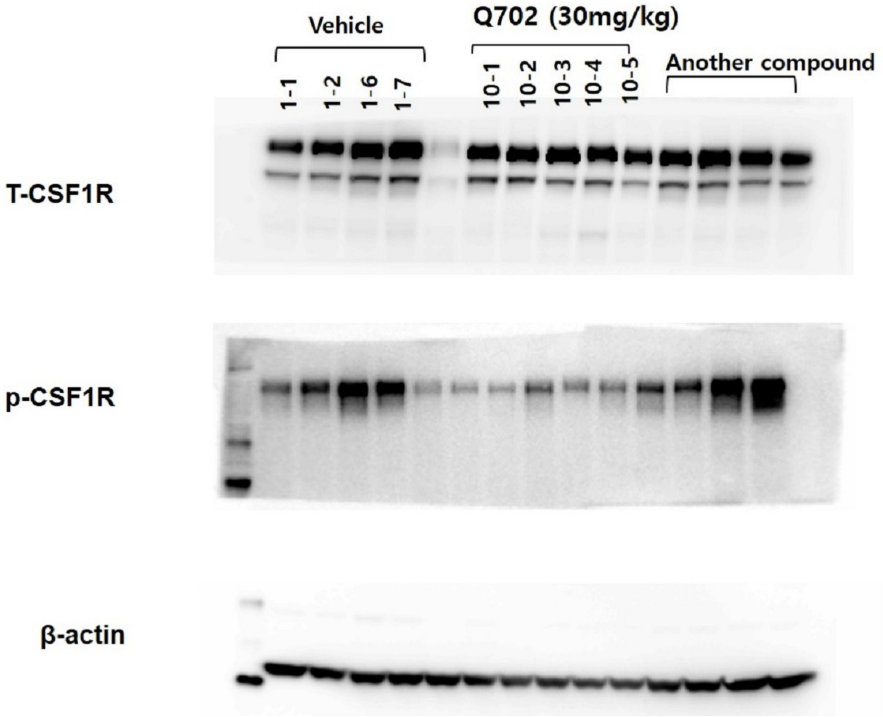

Supplementary Figure S7. Full western blots of Figure 1C, right (M-NFS-60 tumor samples).
